# Study of the Structure-Function-Stability Relationships in Yeast D-amino Acid Oxidase: Hydrophobization of Alpha-Helices

**Published:** 2014

**Authors:** I. V. Golubev, N. V. Komarova, K. V. Ryzhenkova, T. A. Chubar, S. S. Savin, V. I. Tishkov

**Affiliations:** Department of Chemistry, M.V. Lomonosov Moscow State University; Leninskie gory, 1/3, Moscow, 119991, Russia; Innovations and High Technologies MSU Ltd, Tsymlyanskaya str., 16-96, Moscow, 109559, Russia; A.N. Bach Institute of Biochemistry, Russian Academy of Sciences, Leninskiy ave., 33/2, Moscow, 119071, Russia

**Keywords:** D-amino acid oxidase from yeast Trigonopsis variabilis, protein engineering, hydrophobization of alpha-helices, site-directed mutagenesis, substrate specificity, thermal stability

## Abstract

Hydrophobization of alpha-helices is one of the general approaches used for improving the thermal stability of enzymes. A total of 11 serine residues located in alpha-helices have been found based on multiple alignments of the amino acid sequences of D-amino acid oxidases from different organisms and the analysis of the 3D-structure of D-amino acid oxidase from yeast *Trigonopsis variabilis *(TvDAAO, EC 1.4.3.3). As a result of further structural analysis, eight Ser residues in 67, 77, 78, 105, 270, 277, 335, and 336 positions have been selected to be substituted with Ala. S78A and S270A substitutions have resulted in dramatic destabilization of the enzyme. Mutant enzymes were inactivated during isolation from cells. Another six mutant TvDAAOs have been highly purified and their properties have been characterized. The amino acid substitutions S277A and S336A destabilized the protein globule. The thermal stabilities of TvDAAO S77A and TvDAAO S335A mutants were close to that of the wild-type enzyme, while S67A and S105A substitutions resulted in approximately 1.5- and 2.0-fold increases in the TvDAAO mutant thermal stability, respectively. Furthermore, the TvDAAO S105A mutant showed on average a 1.2- to 3.0-fold higher catalytic efficiency with D-Asn, D-Tyr, D-Phe, and D-Leu as compared to the wild-type enzyme.

## INTRODUCTION

D-amino acid oxidase (DAAO, [EC 1.4.3.3]) belongs to a class of FAD-containing oxidoreductases and catalyzes the oxidative deamination of D-amino acids to the corresponding α-keto acids [1]. DAAO is widespread in nature: the genes of this enzyme have been found in cells of molluscs, fishes, reptiles, amphibians, insects, birds, plants, mammals, as well as microorganisms, including fungi, yeasts and bacteria, where it performs important physiological functions [2, 3]. The processes of synthesis of optically active compounds, α-keto acids, and 7-aminocephalosporanic acid using DAAO have been designed. This enzyme is also used in biosensors to determine the D-amino acid content [2, 4, 5]. Two enzymes from yeasts *Rhodotorula gracilis *(RgDAAO) and* Trigonopsis variabilis *(TvDAAO) are the most widely used ones. TvDAAO exhibits the highest activity with cephalosporin C (CephC) [6] and the best thermal stability [7] among all known D-amino acid oxidases. For example, TvDAAO retains 100% activity when incubated for 30 min at 45°C, while RgDAAO is completely inactivated under these conditions. The temperature stability of DAAO from *Arthrobacter protophormiae* and *Candida boidinii *was also studied. They are very similar to RgDAAO. Thus, at 50°C they completely lose their activity in 30 minutes [5, 8, 9].

In our laboratory, the D-amino acid oxidase gene from *T. variabilis *yeast has been cloned, the overexpression system of the recombinant enzyme in *Escherichia coli *cells in soluble and active form has been developed, and its properties have been studied [10]. Native TvDAAO is a homodimer [11], which has a 2-fold symmetry axis with the subunits mutually arranged in a “head-to-tail” manner. Each subunit contains one FAD cofactor molecule at the active site.

Increasing the thermal stability of practically important enzymes is both a fundamental and applied problem. Data produced in such experiments provide a more comprehensive and deeper understanding of the relationship between the structure, function, and stability of the protein being studied. At the same time, solving this problem allows one to reduce the loss of enzyme during its isolation and facilitates the purification process, which in turn reduces the cost of the final product. For example, mutant formate dehydrogenases from *Pseudomonas sp. 101 *with increased temperature stability were obtained in our laboratory. This allowed us to introduce the thermal treatment step to the purification process of the recombinant enzyme. Heating the cell-free extract at 60°C for 20-30 minutes resulted in increased purity of the preparation ranging from 50 to 80-85% without a loss of the enzyme activity [12].

Very scarce data on increasing the thermal stability of TvDAAO using protein engineering have been published so far. Only two papers report data on the obtained TvDAAO mutants with point amino acid substitutions, which demonstrated a slight increase in thermal stability as compared to the wild-type enzyme [13, 14]. The covalent crosslinking of two TvDAAO subunits with Lys-Leu dipeptide [15] was also reported. It resulted in increased T_m_ by 2°C but worse catalytic properties with most substrates.

This paper presents the results of applying the general approach of increasing thermal stability based on hydrophobization to D-amino acid oxidase from the yeast *T.variabilis*. Hydrophobization was achieved by replacing the serine residues with the alanine residues in the α-helical segments of the TvDAAO structure. The effect of these substitutions on the catalytic properties of the enzyme was also studied.

## EXPERIMENTAL

Molecular Biology Grade reagents were used for the genetic engineering experiments. Bacto tryptone, yeast extract and agar (Difco, USA), glycerol (99.9%) and calcium chloride (“ultra pure”), potassium hydrogen phosphate, sodium dihydrogen phosphate (“pure for analysis”), lysozyme (Fluka/BioChemika, Switzerland), isopropyl-β-D-thiogalactopyranoside (IPTG), 2,2’-azino- bis (3-ethylbenzothiazoline-6-sulfonate) (ABTS), kanamycin and chloramphenicol (Sigma, USA), and glucose and sodium chloride (“AR grade”, ”Helicon”, Russia) were used in the microbiological experiments. Restriction endonucleases, DNA ligase of T4 phage, and Pfu-DNA polymerase (Thermo Scientific) were used for cloning DNA fragments and site-directed mutagenesis. Thermo Scientific reagent kits were used to isolate DNA from agarose gel and plasmids from *E. coli *cells. The oligonucleotides for the polymerase chain reaction (PCR) and sequencing were synthesized by Synthol (Russia). The MilliQ (Millipore, USA) purified water was used in these experiments.

We used the following *E. coli *bacterial strains in our study:

*E. coli *DH5α: *fhuA2 *Δ*(argF-lacZ)U169 phoA glnV44 Φ80 *Δ*(lacZ)M15 gyrA96 recA1 relA1 endA1 thi-1 hsdR17.*

*E. coli *BL21(DE3) pLysS Codon Plus: B F^–^*ompT hsdS*(r_B_^-^ m_B_^-^) *dcm*^+^ Tet^r^*gal *λ(DE3) *endA *Hte [pLysS* argU ileY leuW *Cam^r^].

All reagents for the electrophoresis of proteins were manufactured by Bio-Rad (USA). Tris (tris (hydroxymethyl) aminomethane, “reagent grad”) from Merck (Germany), racemic amino acids from Dia-M (Russia), and Reanal (Hungary), 2,2’-azino-bis (3-ethylbenzthiazoline- 6-sulfonate) (ABTS) (Sigma, USA), horseradish peroxidase (Dia-M, Russia) were used for purification and characterization of the enzyme.

**Site-directed mutagenesis**

Nucleotide substitutions were introduced using twostep PCR as described previously [13, 16]. The plasmid obtained based on pET -33b (+) with the *tvdaao *gene being under the control of a strong promoter of RNA polymerase of T7 phage was used as a template. The mutations were introduced using direct (T7_For) and reverse (T7_Rev) primers at the beginning and at the end of the gene, respectively, as well as direct (Mut_ For) and reverse (Mut_Rev) primers carrying the desired replacements for the *tvdaao *gene. The primer sequences are shown below. The introduced mutations are highlighted in bold.

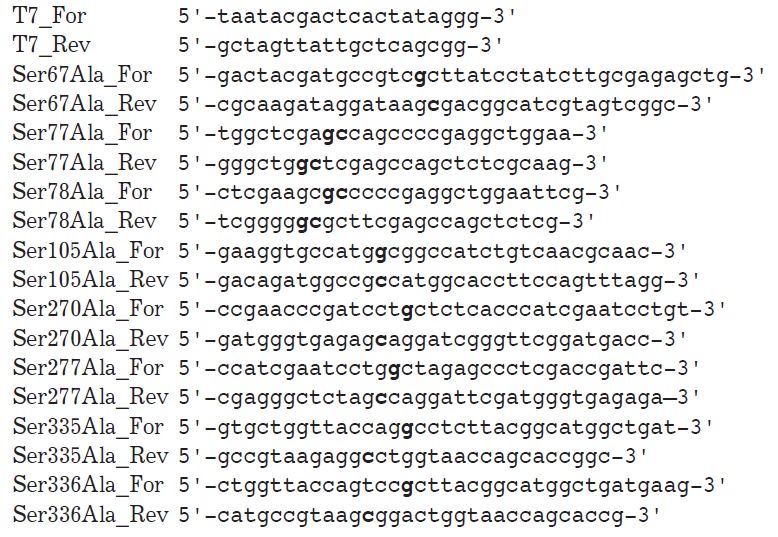



The reaction mixture for PCR contained 2.5 μl of a 10x buffer for Pfu-DNA polymerase (200 mM Tris-HCl (pH 8.8 at 25°C), 100 mM (NH4)2SO_4_, 100 mM KCl, 1 mg/ ml BSA, 1% (v/v) Triton X-100, 20 mM MgSO4); 2.5 μl of a dNTP mixture (dATP, dGTP, dTTP, dCTP with the concentration of each component 2.5 mM); 1 μl of the DNA template (≈10 ng/μL); 2 μl of each primer (10 nM/ml); 0.5 μl of Pfu-DNA polymerase (2.5 U/μl); and deionized water to the total volume of the mixture of 25 μl. PCR was performed in a 0.5-ml thin-walled plastic tube (SSI, USA) using the Tertsik instrument (DNA-Technologies, Russia). A total of 30 μl of mineral oil was added to the tube before the PCR to prevent evaporation of the reaction mixture. The tube was heated for 5 min at 95°C, and the PCR reaction was then initiated by addition of the enzyme. The reaction was done according to the following scheme: the first step at 95°C, 30 sec; the second step at 54-58°C, 60s; and the third step at 72°C, 2 min, a total of 25-35 cycles. After the last cycle, the reaction mixture was further incubated for 10 min at 72°C. The temperature at the second step was 3-5°C below the melting temperature of the duplexes (T_m_) formed by the primers. The empirical formula was used to evaluate T_m_:

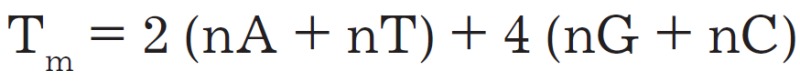



where nX is the number of X-type nucleotides (X = A, T, C, G) in the primer.

Two PCRs using T7_For/Mut_Rev (fragment 1) and Mut_For/T7_Rev (fragment 2) primer pairs were used to obtain fragments containing the desired substitution. The PCR products–fragment 1 and fragment 2–were purified using electrophoresis in a 1% agarose gel. The third uniting PCR was then performed with T7_For and T7_Rev primers, wherein the previously obtained fragments 1 and 2 were used as the DNA template. The product of the third PCR was purified in a similar way using a 1% agarose gel and treated with two restriction endonucleases. NcoI and Bsp119I were used in the case of substitutions at the 67, 77, 78, and 105 positions, while Bsp119I and XhoI were used for substitutions at the 270, 277, 335, and 336 positions. DNA fragments were purified using electrophoresis in a 1% agarose gel and ligated into the original vector, treated with the same restriction endonucleases. The ligation mixture was used to transform *E. coli *DH5α cells. The cells were then plated onto Petri dishes with an agar medium containing kanamycin (30 μg/ml) and incubated for 16 hours at 37°C. Three colonies of each mutant were taken from each plate and used to isolate plasmids. The presence of only desired mutations was proved by sequencing using the plasmid DNA at the center for collective use “Genome” (V.A. Engelhardt Institute of Molecular Biology, Russian Academy of Sciences).

**Expression of TvDAAO mutants in** *E. coli* **cells.**

TvDAAO and its mutants were expressed in *E. coli *cells BL21 (DE3) CodonPlus/pLysS. The cells were transformed using the appropriate plasmid and plated on Petri dishes with an agar medium containing kanamycin (30 μg/ml) to obtain the producer strain. A single colony was taken from the plate and cultured for 16 hours at 30°C in 10 ml of a 2YT medium (Bacto tryptone 16 g/l, yeast extract 10 g/l, sodium chloride 5 g/l, pH 7.5) in the presence of 30 μg/ml kanamycin and 25 μg/ml chloramphenicol to prepare the inoculum. In the morning, the cells were subcultured to a fresh medium (dilution 1: 100) and cultured at 30°C until the absorbance of A600 ≈0.6-0.8 at 600 nm was reached. The inoculum was placed into the culture flasks in amounts of 10% of the total volume of a medium (LB modified medium –yeast extract 10 g/l, Bacto tryptone 5 g/l, glucose 5 g/l, sodium dihydrogen phosphate 1.5 g/l, dipotassium hydrogenphosphate 1 g/l, pH 7.5) containing l kanamycin 30 μg/m. Cultivation was carried out in 1l baffled conical flasks (the volume of the medium did not exceed 10-15% of the flask volume). The cultivation temperature ranged from 18 to 27°C, and the rotation rate of the shaker was 120-160 rpm. After reaching A600 ≈0.6–.8, enzyme expression was induced by adding IPTG to the medium to a final concentration of 0.1 mM. After induction, the cells were cultivated for 24 hours and then pelleted using the Eppendorf 5403 centrifuge (5 minutes, 5000 rpm, 4°C). The resulting pellet was resuspended in a 0.02-M Tris-HCl buffer (pH 8.0 at 25°C) in a ratio of 1: 4 (wt.). The resulting suspension was stored at –20°C.

**Isolation and purification of TvDAAO mutants**

Cell suspension in the 20 mM Tris-HCl buffer with pH 8.0 was twice frozen and thawed, and the cells were then disrupted using a Branson Sonifier 250 (Germany) under continuous cooling to isolate mutant TvDAAO. The precipitate was removed by centrifugation on an Eppendorf 5804 R centrifuge (11000 rpm, 30 min).

Purification of the enzyme included ion exchange chromatography on a MonoQ HR 10/10 column using the FPLC instrument manufactured by Pharmacia Biotech (Sweden) and desalting on a Sephadex G-25 carrier. [17] The purity of the preparations was monitored by analytical electrophoresis in a 12% polyacrylamide gel in the presence of 0.1% sodium dodecyl sulphate on a MiniProtean III instrument (BioRad, Austria) according to the manufacturer’s protocols.

**Kinetic assay**

The activity of D-amino acid oxidase was determined using the bi-enzymatic system, including DAAO and horseradish peroxidase. D-methionine was used as a substrate for the first enzyme, and ABTS was used as a substrate for the second enzyme. The activity was measured at 30°C based on the concentration of the ABTS oxidation product (absorbance at 414 nm, ε_414_ = 36600 l/mol/cm) on a Shimadzu UV-1800 spectrophotometer (Japan). A total of 770 μl of a 50-mM potassium phosphate buffer (PPB), pH 8.0, pre-saturated with air, 200 μl of a 100-mM sodium D-Met solution in 50 mM PPB, 20 μl of a ABTS water solution (16 mg/ml), and 10 μl of a peroxidase solution in 50 mM PPB (5 mg/ml) were added to the spectrophotometer’s cuvette (working volume 1 ml, optical path 1 cm). After incubation for 10 min at 30°C, a sample of wild-type TvDAAO or the corresponding mutant was added to the cuvette (30 μl).

When determining the maximum reaction rate (V_m_) and Michaelis constant (K_M_), the concentration of the corresponding D-amino acid was varied from 0.5 to 5 K_M_. An approximate K_M_ value was determined in a separate experiment by measuring the reaction rate at concentrations of the corresponding D-amino acid of 0.1, 0.5, 1.0, 5.0, 10.0, and 50 mM. The kinetic parameters V_m_ and K_M_ were calculated by nonlinear regression using the OriginPro 8.5 SR1 (OriginLab) program. The catalytic constant k_cat_ was calculated based on the V_m_ value. The concentration of the active enzyme was determined spectrophotometrically based on absorbance at 455 nm using a FAD molar absorption coefficient of 10,800 m^-1^cm^-1^ [6].

**Thermal inactivation study**

The temperature stability of mutant TvDAAO and the wild-type enzyme was studied in a 0.1-M potassium phosphate buffer, pH 8.0. A series of 0.5 ml plastic test tubes containing 100 μl of the enzyme solution were prepared for each experiment. The tubes were placed to a preheated to the desired temperature water thermostat (temperature control accuracy ± 0.1°C). The test tubes were sampled one by one after fixed time intervals, rapidly cooled for 1-2 min in ice, and the enzyme activity was measured as described above. The sampling interval was adjusted to achieve a decrease in the enzyme activity to 10-15% of the baseline value during the experiment. The time dependence of the residual activity of the enzyme was plotted in semilogarithmic coordinates and processed using the OriginPro 8.5 SR1 (OriginLab) program as described in [18] to calculate the inactivation rate constant.

**Computer simulation**

Analysis of the TvDAAO structure, computer simulation of TvDAAO with amino acid mutations, and visualization of the protein globule was performed using the Accelrys Discovery Studio 2.1 software package.

## RESULTS AND DISCUSSION

**Selection of amino acid residues for site-directed muthagenesis**

Rational protein design is a powerful method for studying the structure-function relationships and side-directed changes in an enzyme’s properties. Comparison of the amino acid sequences of the enzymes of interest and the enzymes from thermophilic organisms, as well as analysis of the three-dimensional structure (if it is available for at least one enzyme in the family) is used for directional increase of thermal stability of the enzymes by means of identifying the amino acid residues that play an important role in the stability [19]. However, this approach is not applicable in the case of TvDAAO, since it is the most stable enzyme among the presently studied D-amino acid oxidases, and no amino acid sequences of DAAO from thermophilic microorganisms have been identified so far. Therefore, we decided to use one of the common approaches based on the hydrophobization of the α-helices in the enzyme’s structure [19, 20] to improve the thermal stability of TvDAAO. This can be achieved using various substitutions, e.g., Ser → Ala (most frequently used), Lys → Arg, Gly → Ala, Ser → Thr, Lys → Ala, Thr → Ala, Lys → Glu, Glu → Arg, and Asp → Arg [21]. Ser → Ala substitution usually gives the highest stabilizing effect. For example, the hydrophobization of α-helices by means of Ser → Ala substitution was used to increase the temperature stability of the formate dehydrogenase from* Pseudomonas sp. 101 *[22].

**Fig. 1:**
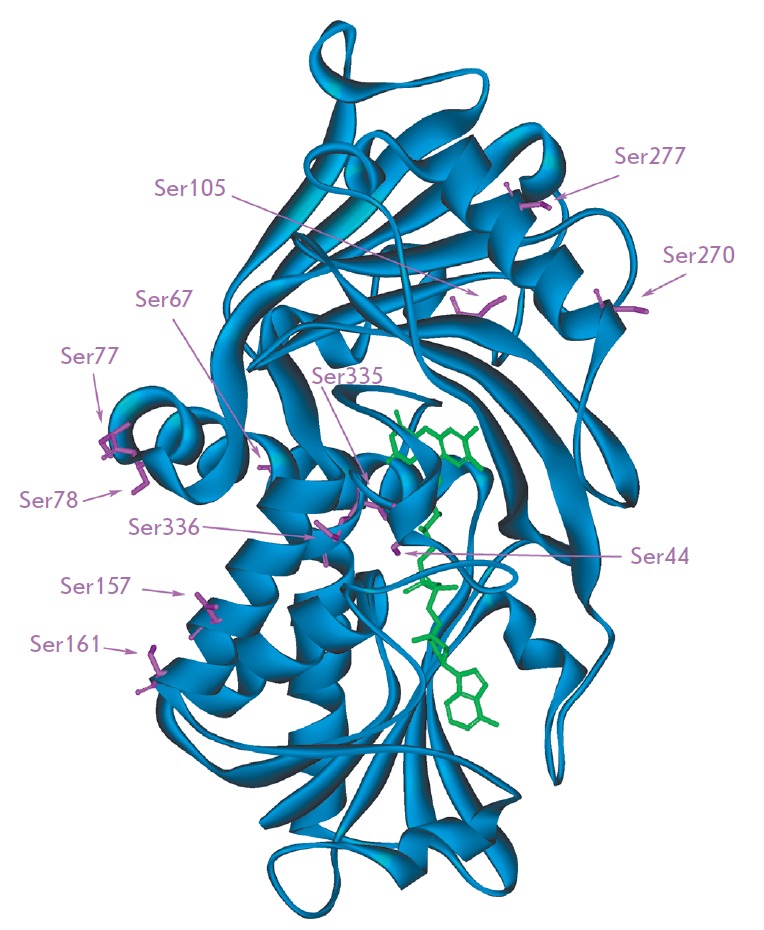
General view of one subunit of TvDAAO with Ser residues located in alpha-helices

**Fig. 2:**
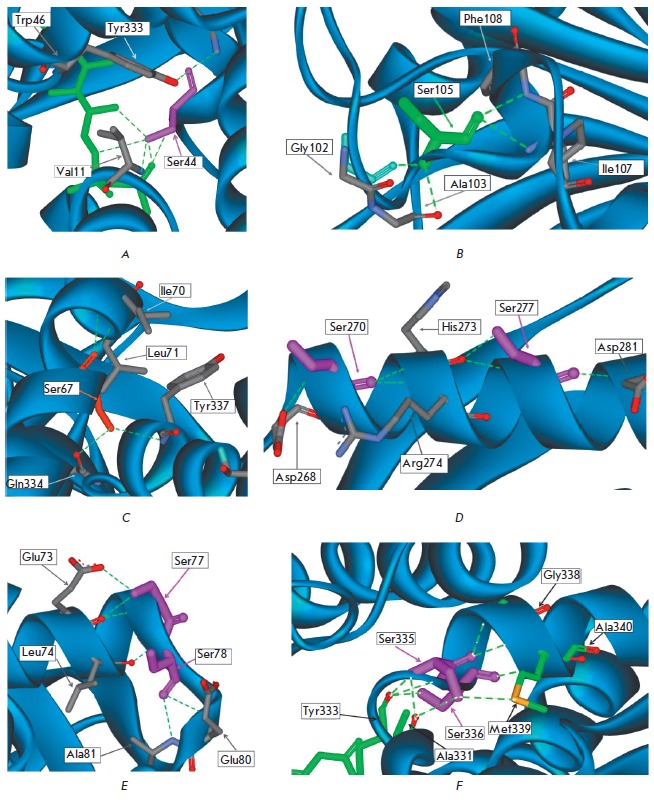
Location and interactions of the Ser residues in alpha-helices in TvDAAO globule

We have analyzed the 3D-structure of TvDAAO to identify potential Ser residues in α-helices. The following points were taken into account when selecting the Ser residues that can be replaced by Ala: 1) the residues should be part of α-helices; 2) and they should not be conserved as well as located at the active site of the enzyme. The analysis of the TvDAAO structure has revealed a total of Ser 11 residues in the α-helices (*Fig. 1*). Comparison of DAAO amino acid sequences from different sources has shown that the Ser44 residue is conserved. It is located in the cofactor- binding domain of TvDAAO, and its side chain forms two hydrogen bonds with the FAD molecule, as established by computer analysis (*Fig. 2A*). Therefore, this residue was excluded from the list of potential replacement candidates. The Ser157 and Ser161 residues are located at the intersubunit area. Therefore, replacement of these residues is also undesirable, despite the fact that they do not participate in the formation of intersubunit hydrogen bonds [11]. Thus, eight Ser residues were selected to be replaced with Ala residues (positions 67, 77, 78, 105, 270, 277, 335, and 336). The Ser67, Ser105, Ser335, and Ser336 residues are located inside the protein globule, while Ser77, Ser78, Ser270, and Ser277 are exposed to the solution. *Fig. 2B-F* shows the position of the selected residues in more detail. Ser67 is located in the middle, while the Ser77 and Ser78 residues are at the end of α3-helix. Ser105 is located in the short α4-helix, and Ser270 and 277 are located at the beginning and in the middle of the α9-helix, respectively. The Ser335 and Ser336 residues are located at the beginning of the α13-helix. All eight serine residues form two to six hydrogen bonds. Ser78, Ser105, and Ser270 form hydrogen bonds with other amino acid residues of the polypeptide chain including only the atoms involved in the peptide bonds. Since the side chains of these serines residues are not involved in hydrogen bonding with other amino acids, the replacement of these three residues with Ala should not result in loosing of hydrogen bonds. The Ser residues at positions 67, 77, 277, 335, and 336 form hydrogen bonds both with the peptide bond atoms and with the side chain hydroxy-groups of other amino acids (see *Fig. 2*). On the one hand, the substitution of these five serine residues will result in a loss of the hydrogen bonds formed by the side chains, which can negatively affect the stability of the enzyme, but on the other hand, increased hydrophobicity of the α-helix can stabilize the protein globule, so that the total effect will be positive. Therefore, the replacement of these serine residues is of theoretical interest in terms of the influence of these two factors on the stability of TvDAAO.

**Preparation of TvDAAO mutants with Ser / Ala substitutions**

**Fig. 3:**
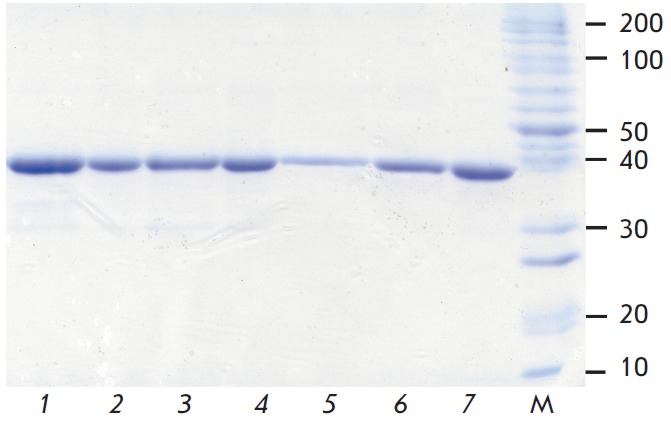
Analysis by 12% SDS-PAGE of soluble mutant TvDAAOs with amino acid changes: 1 – Ser67Ala, 2 – Ser77Ala, 3 – Ser105Ala, 4 – Ser277Ala, 5 – Ser335Ala, 6 – Ser336Ala and 7 – wild-type enzyme. M – molecular- mass size marker

The nucleotide substitutions in the *tvdaao *gene that resulted in the desired mutation were introduced using PCR. Three plasmids were sequenced for each of the eight mutant *tvdaao *genes. It has been shown that in all cases only the desired mutations in the *tvdaao* gene were present and that there were no other nucleotide changes. Plasmids with mutated TvDAAO genes were used to transform *E. coli *BL21 (DE3) Codon Plus / pLysS cells. The resulting recombinant strains were cultivated as described in the Experimental section. All eight TvDAAO mutants were synthesized in a soluble form and demonstrated enzymatic activity. Two TvDAAO mutants with Ser78Ala and Ser270Ala substitutions could not be obtained in the purified form, as they were rapidly inactivated during cell disruption, which is indicative of strong destabilization of the protein globule. The remaining six TvDAAO mutants with Ser67Ala, Ser77Ala, Ser105Ala, Ser277Ala, Ser335Ala, and Ser336Ala substitutions were isolated and purified using anion exchange chromatography. Their purity was at least 99% according to the results of analytical electrophoresis in a polyacrylamide gel in the presence of sodium dodecyl sulfate (see *Fig. 3*, lanes 1-6).

**Catalytic properties of TvDAAO mutants**

**Fig. 4:**
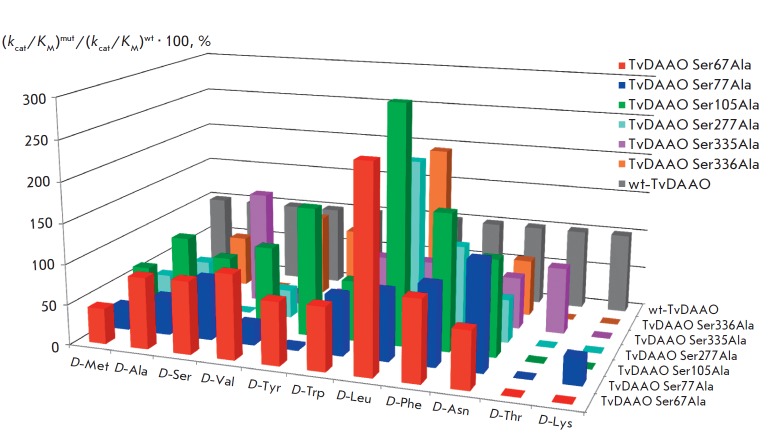
Relative catalytic efficiencies ((kcat/KM)mut/(kcat/KM)wt*100%) of mutant TvDAAOs with Ser67Ala, Ser77Ala, Ser105Ala, Ser277Ala, Ser335Ala, and Ser336Ala substitutions. The catalytic efficiency of the wild-type TvDAAO is taken as 100%

The Michaelis constant (K_M_) and catalytic constant (k_cat_) with various D-amino acids were determined for the six TvDAAO mutants, including Ser67Ala, Ser77Ala, Ser105Ala, Ser277Ala, Ser335Ala, and Ser336Ala substitutions. The values of k_cat_, K_M_ and catalytic efficiency k_cat_/K_M_ of the TvDAAO mutants and the wild-type enzyme with various D-amino acids are shown in *Table 1*. The improvement of the kinetic parameter as compared to that of the wild-type enzyme is shown in bold on a gray background. For clarity, *Fig. 4* shows the catalytic efficiency values

The following points in relation to the individual mutants should be emphasized:

1. The properties of Ser67Ala TvDAAO are similar to those of the wild-type enzyme with many substrates. A significant increase in the catalytic efficiency (2.5 fold) was observed only with D-Leu. The enzyme was inactive with D-Thr and D-Lys.

2. Ser77Ala TvDAAO shows higher catalytic efficiency only with D-Asn. The enzyme is inactive with D-Thr, and the activity with D-Tyr, D-Met, and D-Val is significantly decreased. Only this mutant retained its activity with D-Lys.

3. Ser105Ala TvDAAO has the best catalytic parameters between all mutant forms, except for the lack of activity with D-Thr and D-Lys. The catalytic efficiency decreased by 1.3-fold with D-Trp and 1.6-fold with D-Met, but it increased by 1.6-, 1.7-, and 3.0-fold with D-Tyr, D-Phe and D-Leu, respectively.

4. Ser335Ala TvDAAO has a higher catalytic activity with D-Ser as compared to that of the wild-type enzyme. Moreover, only this mutant enzyme retained its activity with D-Thr.

**Fig. 5:**
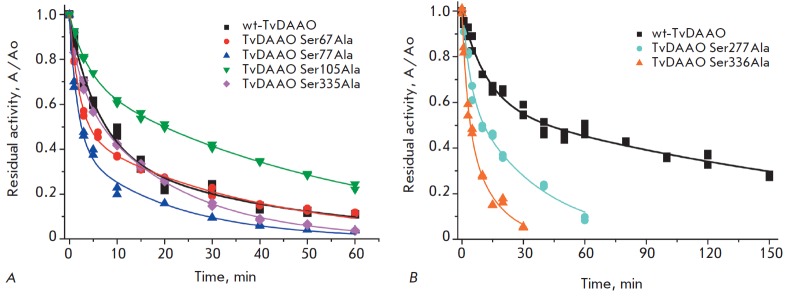
A – Time dependence of the residual activity of the wild-type and mutant TvDAAOs S67A, S77A, S105A, and S335A at 56°C. 0.1 M potassium phosphate buffer, pH 8.0. Enzyme concentration is 10 μg/ml B – Time dependence of the residual activity of the wild-type and mutant TvDAAOs S277A and S336A at 52°C. 0.1 M potassium phosphate buffer, pH 8.0. Enzyme concentration is 10 μg/ml

Unlike the other enzymes, the Ser277Ala and Ser- 336Ala TvDAAO mutants were characterized by a complete loss of activity with D-Ser, along with retained activity with D-Ala, which can be used for the selective detection of D-Ala in biological samples in the presence of D-Ser. These enzymes are also inactive with D-Thr and D-Lys, but they exhibit higher catalytic efficiency with D-Leu.

**Table 1:** Catalytic properties of mutant TvDAAOs and wild-type on D-amino acids

D-amino acid	Enzyme											
	wt-TvDAAO			TvDAAO Ser67Ala			TvDAAO Ser77Ala			TvDAAO Ser105Ala		
	k_cat_, s^-1^	K_M_, mM	k_cat_/ K_M_, mM^-1^s^-1^	k_cat_, s^-1^	K_M_, mM	k_cat_/ K_M_, mM^-1^s^-1^	k_cat_, s^-1^	K_M_, mM	k_cat_/ K_M_, mM^-1^s^-1^	k_cat_, s^-1^	K_M_, mM	k_cat_/ K_M_, mM^-1^s^-1^
D-Met	80.5 ± 0.8	0.46 ± 0.03	175	104 ± 3.0	1.35 ± 0.04	77.4	44.0 ± 2.0	0.85 ± 0.09	52.0	153 ± 2.0	1.38 ± 0.04	110
D-Ala	108.6 ± 2.0	16.7 ± 0.7	6.5	180 ± 13	31.0 ± 4.0	5.8	33.7 ± 1.0	11.1 ± 0.1	3.1	218 ± 8.0	31.3 ± 2.0	7.0
D-Ser	20.5 ± 0.9	36.6 ± 3.3	0.56	18.4 ± 0.7	36.0 ± 3.0	0.51	7.9 ± 0.2	18.3 ± 0.8	0.43	10.4 ± 0.4	21.4 ± 1.9	0.49
D-Val	85.3 ± 2.7	14.4 ± 1.2	5.9	133 ± 6.0	21.4 ± 1.6	6.2	19.1 ± 0.5	12.2 ± 0.8	1.56	154 ± 5.0	24.7 ± 1.3	6.3
D-Tyr	22.5 ± 1.9	0.45 ± 0.06	50.0	34.0 ± 4.0	0.87 ± 0.14	38.9	12.4 ± 0.8	6.5 ± 0.7	1.90	50.0 ± 7.0	0.63 ± 0.12	80.0
D-Trp	42.4 ± 1.4	0.49 ± 0.04	86.5	35.9 ± 1.3	0.53 ± 0.4	67.7	43.7 ± 0.8	0.68±0.03	64.3	38.6 ± 1.6	0.60 ± 0.05	64.6
D-Leu	29.1 ± 0.3	0.78 ± 0.02	37.3	31.4 ± 0.5	0.34 ± 0.02	93.1	6.2 ± 0.5	0.20 ± 0.04	31.9	30.4 ± 0.4	0.28 ± 0.02	110
D-Phe	27.2 ± 0.8	0.37 ± 0.04	73.9	30.4 ± 1.2	0.41 ± 0.04	73.6	23.4 ± 0.5	0.32 ± 0.03	73.1	32.3 ± 0.6	0.26 ± 0.02	124.3
D-Asn	62.4 ± 2.0	22.6 ± 1.5	2.8	49.7 ± 1.7	25.7 ± 1.5	1.94	17.2 ± 1.0	4.7 ± 0.7	3.7	48.3 ± 1.2	14.5 ± 0.7	3.3
D-Thr	1.75 ± 0.04	11.1 ± 0.8	0.16	no reaction			no reaction			no reaction		
D-Lys	3.54 ± 0.21	29.3 ± 3.4	0.12	no reaction			< 1.2	> 50	0.04	no reaction		

**Table:** Table 1 part 2

D-amino acid	Enzyme								
	TvDAAO Ser277Ala			TvDAAO Ser335Ala			TvDAAO Ser336Ala		
	k_cat_, s^-1^	K_M_, mM	k_cat_/ K_M_, mM^-1^s^-1^	k_cat_, s^-1^	K_M_, mM	k_cat_/ K_M_, mM^-1^s^-1^	k_cat_, s^-1^	K_M_, mM	k_cat_/ K_M_, mM^-1^s^-1^
D-Met	48.0 ± 1.0	0.73 ± 0.04	65.8	45.9 ± 1.1	4.6 ± 0.2	9.9	56.8 ± 1.5	2.69 ± 0.13	21.1
D-Ala	81.0 ± 3.0	20.4 ± 1.4	4.0	8.2 ± 0.4	10.1 ± 1.2	0.82	101 ± 5.0	24.2 ± 1.9	4.2
D-Ser	no reaction			34.1 ± 1.7	43.0 ± 5.0	0.79	no reaction		
D-Val	71.0 ± 3.0	34.0 ± 3.0	2.1	20.1 ± 1.3	25.0 ± 3.0	0.82	50.0 ± 2.0	8.2 ± 0.9	6.1
D-Tyr	0.45 ± 0.06	0.70 ± 0.14	0.65	5.5± 0.9	0.58 ± 0.13	9.5	15.8 ± 0.7	0.36 ± 0.03	44.2
D-Trp	0.49 ± 0.04	0.26 ± 0.02	1.9	79.0 ± 5.0	1.23 ± 0.11	64.1	57.0 ± 1.0	0.69 ± 0.04	82.0
D-Leu	12.0 ± 2.0	0.15 ± 0.02	80.0	5.9 ± 0.1	0.21 ± 0.01	27.5	5.0 ±0.1	0.07 ± 0.01	76.4
D-Phe	15.1 ± 0.4	0.18 ± 0.03	83.9	8.3 ± 0.2	0.38 ± 0.03	21.9	11.8 ± 0.3	0.24 ± 0.02	49.4
D-Asn	11.8 ± 1.2	8.0 ± 1.5	1.48	46.0 ± 4.0	26.0 ± 3.0	1.78	32.0 ± 2.0	16.0 ± 3.0	2.0
D-Thr	no reaction			1.8 ± 0.1	14.0 ± 2.0	0.13	no reaction		
D-Lys	no reaction			no reaction			no reaction		

^*^ Improved catalytic parameters of mutant TvDAAOs in comparison to wild-type are marked with bold font and gray background

## TEMPERATURE STABILITY OF TVDAAO MUTANTS

**Stability of Ser78Ala and Ser270Ala TvDAAO**

As noted above, Ser78Ala and Ser270Ala substitutions led to strong destabilization of the protein globule, so that the enzymes were inactivated during their isolation from the cells. Computer simulations have shown that the side chains of Ser78 and Ser270 do not form hydrogen bonds with neighboring residues. However, they are located in the immediate vicinity of the Glu80 and Asp268 residues, respectively, which can form hydrogen bonds in solution, both directly and through a water molecule, since in both cases the distance between the hydroxy group of serine and the carboxyl group is about 4 A. Ser78 and Ser270 are located on the bends at the end of the α3-helix and at the beginning of the α9-helix, respectively, and therefore they appear to play an important role in maintaining the stability of TvDAAO secondary structure elements, as evidenced by the strong destabilization upon their replacement by alanine residues.

**Stability of Ser67Ala, Ser77Ala, Ser105Ala, Ser277Ala, Ser335Ala, and Ser336Ala TvDAAO mutants**

*Fig. 5 A, B* shows the time dependence of the residual activity of the TvDAAO mutants at the same concentration. As it can be seen from *Fig. 5A*, Ser77Ala and Ser335Ala substitutions result in slightly reduced stability. Ser67Ala substitution does not affect the stability of the enzyme, while Ser105Ala substitution results in noticeable stabilization. The most significant destabilization of the protein globule is observed in the case of Ser277Ala and Ser336Ala substitutions (*Fig. 5B*). The incubation temperature had to be reduced from 56 to 52°C to obtain inactivation curves comparable to those of the other TvDAAO mutants.

## MECHANISM OF INACTIVATION OF TVDAAO MUTANTS

We showed [11, 13, 16] that inactivation of wild-type TvDAAO and its various mutants at elevated temperatures proceeds according to the following dissociative mechanism:

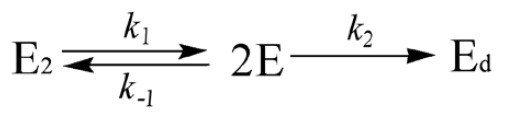



According to this mechanism, the first step includes reversible dissociation of the E_2_ active dimer to form two inactive monomers E. Irreversible transition of E to the denatured monomer E_d_ then occurs. This mechanism was analyzed in details by O.I. Poltorak *et al*. [18]. The time dependence of the residual activity of the enzyme in this mechanism is described by a sum of two exponential functions, and the inactivation rate of the enzyme depends on its concentration [11, 13, 16]. The dissociative mechanism of the wild-type TvDAAO thermoinactivation is only observed within a temperature range of 50-60°C, when the rate constants k_1_ and k_2_ are comparable to each other. The rate constant k_1_ increases more rapidly than the rate constant k_2_ upon increasing temperature; therefore, the first and second steps become the limiting ones at temperatures below 50 and above 60°C, respectively, and the kinetics of inactivation is described by a single exponential function under these conditions, similarly to that of unimolecular reactions.

**Fig. 6:**
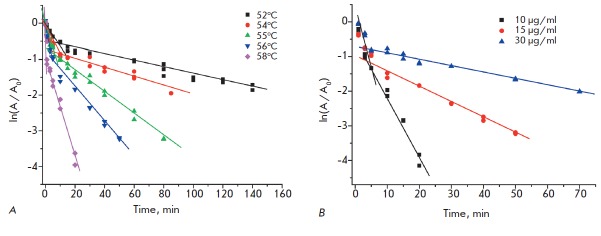
Time dependence of the residual activity of mutant TvDAAO S77A in semi-logarithmic coordinates. A – thermal inactivation at different temperatures and fixed enzyme concentration of 15 μg/ml, B – thermal inactivation at different enzyme concentrations and temperature of 56°C. 0.1 M potassium phosphate buffer, pH 8.0

Analysis of the time dependence of the residual activity shows that the thermal inactivation mechanism of the Ser67Ala, Ser77Ala, Ser105Ala, Ser335Ala TvDAAO mutants (*Fig. 5A*) and Ser277Ala and Ser- 336Ala TvDAAO mutants (*Fig. 5B*) also does not differ from that of the wild-type enzyme. As an example, *Fig. 6 A, B* shows the residual activity of Ser77Ala TvDAAO mutant vs incubation time in semilogarithmic coordinates at various temperatures and concentrations. Similar dependences were obtained for all other mutant enzymes. The presence of the break points on the thermal inactivation curves in semilogarithmic coordinates at different temperatures and increase in the slope of the second linear section along with a decrease in the initial concentration of the enzyme provides evidence that thermal inactivation occurs through a dissociative mechanism [18]. We have calculated the rate constants of the thermal inactivation of the Ser67Ala, Ser77Ala, Ser105Ala, Ser277Ala, Ser335Ala, and Ser336Ala TvDAAO mutants for both stages of the process on the basis of the experimental dependence of the residual enzyme activity vs incubation time (*Table 2*).

**Table 2:** Kinetic parameters of dissociative thermal inactivation of mutant and wild-type TvDAAOs

Enzyme	Parameter	Temperature, °C							
		46	48	50	52	54	56	58	60
TvDAAO Ser67Ala	k_1_∙10^4^, s^-1^	-	-	-	4.45	7.8	14.4	27.4	41.8
	k_2_∙10^4^, s^-1^	-	-	-	2.51	3.25	4.44	5.7	9.6
TvDAAO Ser77Ala	k_1_∙10^4^, s^-1^	-	-	-	3.92	8.3	15.1	26.7	-
	k_2_∙10^4^, s^-1^	-	-	-	2.31	4.20	7.9	18.5	-
TvDAAO Ser105Ala	k_1_∙10^4^, s^-1^	-	-	-	2.43	3.37	5.4	12.6	21.9
	k_2_∙10^4^, s^-1^	-	-	-	2.48	3.44	5.8	7.2	9.7
TvDAAO Ser277Ala	k_1_∙10^4^, s^-1^	3.02	5.4	10.2	14.4	25.9	-	-	-
	k_2_∙10^4^, s^-1^	0.93	1.41	3.25	3.91	7.6	-	-	-
TvDAAO Ser335Ala	k_1_∙10^4^, s^-1^	-	-	3.47	4.80	6.8	10.3	17.7	-
	k_2_∙10^4^, s^-1^	-	-	2.43	3.23	6.1	8.9	17.9	-
TvDAAO Ser336Ala	k_1_∙10^4^, s^-1^	2.88	6.8	13.8	28.4	-	-	-	-
	k_2_∙10^4^, s^-1^	0.90	3.67	9.3	18.5	-	-	-	-
wt-TvDAAO	k_1_∙10^4^, s^-1^	-	-	2.86	3.67	9.2	11.6	21.5	40.5
	k_2_∙10^4^, s^-1^	-	-	2.28	3.13	5.6	7.1	10.8	19.4

^*^ The decrease in inactivation rate constants of mutants as compared to the wild-type enzyme is marked with a green background; the increase is marked with a red background. Different shades show the extent of the effects – a greater effect corresponds to the darker color.

Ser277Ala and Ser336Ala mutations in TvDAAO resulted in a shift in the temperature range associated with the dissociative mechanism by 4°C towards lower temperatures as compared to that of the wild-type enzyme. The Ser336Ala TvDAAO mutant was the least stable of all the mutants that were obtained and purified (see *Fig. 5B*, *Table 2*). At a temperature of 52°C (corresponding kinetic curves are shown in *Fig. 5B*), Ser- 277Ala and Ser336Ala mutations led to 3.9- and 7.7-fold increases in the first-step inactivation rate constants and 1.2- and 5.9-fold increases in the second-step inactivation rate constants, respectively (*Table 2*). Therefore, Ser336Ala mutation results in a destabilizing effect associated with both the first step of inactivation (enzyme dissociation into monomers), and the second step (denaturation of the protein globule). Ser277Ala substitution leads mainly to an increase in the first-step inactivation rate, but the effect is not as significant as that associated with Ser336Ala substitution. The higher effect of the enzyme destabilization associated with Ser336Ala substitution may be due to the fact that the Ser336 residue is located at the end of the α13-helix and forms a hydrogen bond with the peptide bond of the Tyr333 residue, which, in turn, is in close contact with the *Si*-side of the isoalloxazine cycle of FAD and conserved residue Ser44 (*Fig. 2F*). In addition, Ser336 and Tyr333 occur in corresponding positions of the D-amino acid oxidases being the most homologous to TvDAAO, and apparently they play an important role in maintaining the conformation required for cofactor binding. The Ser277 residue is located in the middle of the α9-helix on the surface of the enzyme (*Fig. 2D*) and forms a hydrogen bond with the carbonyl oxygen atom of the peptide bond of the His273 residue. The loss of this hydrogen bond could have a negative effect on the thermal stability of TvDAAO.

The temperature range, in which inactivation of the enzyme associated with substitutions of four Ser residues (67, 77, 105 and 335) occurs through the dissociative mechanism, remained the same, but the values of the thermal inactivation rate constants changed as compared to those of the wild-type enzyme (*Table 2*).

**Fig. 7:**
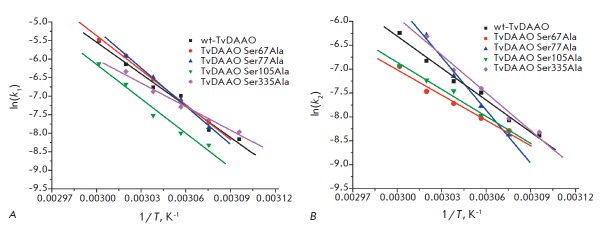
Temperature dependences of the inactivation rate constants of the first (A) and second (B) stages for mutant TvDAAOs with S67A, S77A S105A, S335A mutations and wild-type TvDAAO in semi logarithmic coordinates ln(*k*_in_)* vs *1/T. 0.1 M potassium phosphate buffer, pH 8.0

As mentioned above, the temperature dependences of the first- and second-stage rate constants differ. *Fig. 7 A, B* shows the dependence of ln(k) vs 1/T for the first- and second-stage rate constants, respectively.

Ser105Ala substitution results in a higher stability of the enzyme throughout the whole temperature range of 52 to 60°C (*Table 2* , *Fig. 5A*). The dependences of the thermal inactivation rate constants for both stages are very close to those of the wild-type enzyme, but they are lower on the respective charts over the range from 52 to 60°C, as shown below (*Fig. 7 A, B*). Ser105Ala substitution on average led to two-fold higher thermal stability of TvDAAO as compared to that of the wild-type enzyme at the first stage of thermal inactivation and 1.5- fold higher stability at the second stage, which is rather significant for this enzyme. A similar stabilizing effect was observed at the second stage of inactivation of the Ser67Ala TvDAAO mutant (about 1.6-fold), and the temperature dependence of the inactivation rate constant is also close to that of the wild-type enzyme, but the parameters of the first stage of the thermal inactivation of Ser67Ala TvDAAO are slightly inferior to those of the wild-type enzyme over the entire temperature range; i.e., the stability decreased by by 20% on average. Nevertheless, this substitution also results in overall stabilization of the enzyme, although this stabilization is lower than in the case of Ser105Ala substitution. The Ser67 and Ser105 residues are located inside the protein globule in the middle of the α3-helix and short α4-helix. The hydroxy-group of Ser105 residue forms no hydrogen bonds with neighboring residues and is adjacent to the hydrophobic residues Leu100, Ala103, and Ile107, while the hydroxy-group of Ser67 forms two hydrogen bonds with the polypeptide chain atoms of the Gln334 and Tyr337 residues, and it is also located in close vicinity to the hydrophobic residues Trp51, Leu70, Leu71, and the benzene ring of the Tyr337 residue. Ser105Ala substitution facilitates hydrophobic interactions within the protein globule without breaking any hydrogen bonds, which probably leads to an increase in the thermal stability of TvDAAO. Furthermore, the three dimensional structure of the enzyme may apparently undergo some conformational changes resulting in the stabilization of the dimer and improvement of its catalytic properties. By contrast, Ser67Ala substitution results in the loss of two hydrogen bonds with the Tyr337 and Gln334 residues, which are located in the spatially close α13-helix, but could result in stronger hydrophobic interactions, which contributes to stabilization of the protein globule, as evidenced by the results of experiments.

Ser77Ala and Ser335Ala TvDAAO mutants differ from the rest of mutants in the temperature dependences of the rate constants of the first and the second stages of inactivation (*Fig. 7 A, B*). The first- and the second-stage rate constants of the Ser77Ala mutant increase more rapidly with increasing temperature than those of the wild-type enzyme, and the dependence is more significant in the case of k_2_ (*Fig. 7B*), which results in a lower overall stability of Ser77Ala TvDAAO at temperatures above 54°C, whereas at lower temperatures this mutant is more stable than the wild-type enzyme. In case of Ser335Ala TvDAAO, the temperature dependences of the inactivation rate constants k_1_ and k_2_ oppositely differ from those of the wild-type enzyme. Increase in k_1_ with temperature is less pronounced, while k2 is more strongly temperature-dependent. As a result, Ser335Ala TvDAAO is more stable than the wild-type enzyme at the first stage of inactivation at temperatures > 54°C; and at the second stage, at temperatures < 50°C. Thus, due to the complex temperature dependence of the constants of both inactivation stages, the stability of Ser335Ala TvDAAO at each temperature is given by the ratio of the constants of each inactivation stage. Nevertheless, the overall stability only slightly differs from that of the wild-type enzyme.

## CONCLUSIONS

The effect of hydrophobization of α-helices in the structure of the D-amino acid oxidase from yeast* Trigonopsis variabilis *was studied by replacing eight serine residues with alanine residues. From the viewpoint of the structure-stability relationship, it is interesting that replacement of Ser residues on the surface of TvDAAO at positions 77, 78, 270, and 277 results in destabilization of the enzyme, while replacement of Ser 67, 105, 335, and 336 inside the protein globule leads to a reduced stability only in one case out of four. It should also be noted that replacement of the serine residues located at the ends of α-helices also negatively affects the thermal stability of the enzyme. These data are directly in contradiction to the results obtained for the formate dehydrogenase from the *Pseudomonas sp. 101* bacteria [22]. The highest enzyme stabilization effect (1.6-fold) was observed upon replacing the Ser131 located on the surface of the protein globule, with the Ala residue. Furthermore, stabilization effect was also observed upon replacing Ser184, which is located at the end of the α6-helix [22]. We therefore can conclude that, despite the generality of the approach based on hydrophobization of α-helices, the value and effect of stabilization depend directly on the structural features of the particular protein or enzyme.

In conclusion, we would like to mention that there are cases in protein engineering when a single amino acid substitution results in a significant stabilization of the enzyme [23, 24]. However, usually the improvement of thermal stability can be achieved by combining several successful point mutations. Each of these individual mutations has a moderate stabilization effect, while the temperature stability of a multipoint mutant enzyme becomes significant [12]. Thus, the method of hydrophobization of α-helices cannot be considered as the basic one, but rather as the additional approach to improving the stability of enzymes, due to the low stabilization effects of point amino acid mutations.
